# Enhancing prognostic accuracy: a SEER-based analysis for overall and cancer-specific survival prediction in cervical adenocarcinoma patients

**DOI:** 10.1007/s00432-023-05399-2

**Published:** 2023-09-25

**Authors:** Linlin Chen, Yu Chen, Haoting Shi, Rong Cai

**Affiliations:** 1 Department of Radiation Oncology, Ruijin Hospital, Shanghai Jiaotong University School of Medicine, Shanghai, China; 2 Department of Oncology, Cancer Hospital Affiliated to Guizhou Medical University, Guizhou, China

**Keywords:** Cervical adenocarcinoma, Prognostic nomogram, Overall survival, Cancer-specific survival, SEER

## Abstract

**Background:**

Cervical adenocarcinoma (CA) is the second most prevalent histological subtype of cervical cancer, following cervical squamous cell carcinoma (CSCC). As stated in the guidelines provided by the National Comprehensive Cancer Network, they are staged and treated similarly. However, compared with CSCC patients, CA patients are more prone to lymph node metastasis and recurrence with a poorer prognosis. The objective of this research was to discover prognostic indicators and develop nomograms that can be utilized to anticipate the overall survival (OS) and cancer-specific survival (CSS) of patients diagnosed with CA.

**Methods:**

Using the Surveillance, Epidemiology, and End Result (SEER) database, individuals with CA who received their diagnosis between 2004 and 2015 were identified. A total cohort (*n* = 4485) was randomly classified into two separate groups in a 3:2 ratio, to form a training cohort (*n* = 2679) and a testing cohort (*n* = 1806). Overall survival (OS) was the primary outcome measure and cancer-specific survival (CSS) was the secondary outcome measure. Univariate and multivariate Cox analyses were employed to select significant independent factors and Least Absolute Shrinkage and Selection Operator (LASSO) Cox regression analysis was utilized to develop predictive nomogram models. The predictive accuracy and discriminatory ability of the nomogram were assessed by employing metrics such as the calibration curve, receiver operating characteristic (ROC) curve, and the concordance index (C-index).

**Results:**

Age, Tumor Node Metastasis stages (T, N, and M), SEER stage, grade, and tumor size were assessed as common independent predictors of both OS and CSS. The C-index value of the nomograms for predicting OS was 0.832 (95% CI 0.817–0.847) in the training cohort and 0.823 (95% CI 0.805–0.841) in the testing cohort.

**Conclusion:**

We developed and verified nomogram models for predicting 1-, 3- and 5-year OS and CSS among patients with cervical adenocarcinoma. These models exhibited excellent performance in prognostic prediction, providing support and assisting clinicians in assessing survival prognosis and devising personalized treatments for CA patients.

**Supplementary Information:**

The online version contains supplementary material available at 10.1007/s00432-023-05399-2.

## Introduction

Cervical cancer is the fourth most prevalent malignancy among women globally (Small et al. 2017). In 2020, the incidence of cervical cancer was approximately 13.3 per 100,000 females, and mortality was 7.2 per 100,000 females, with higher rates among young women aged 20–39 years (roughly nine deaths per week) (Cohen et al. 2019; Siegel et al. 2019). Cervical adenocarcinoma (CA), accounting for approximately 10–25% of all cervical cancer cases, is the second most prevalent subtype after cervical squamous cell carcinoma (CSCC) (Rivera-Colon et al. 2020). However, as cervical screening methods improve, particularly among young women under 40, CSCC rates are declining while CA rates are increasing (Bray et al. 2005). Moreover, CA patients are more likely to experience lymph node metastasis and recurrence with a worse prognosis (Irie et al. 2000).

The current approaches to treatment and factors influencing prognosis for cervical cancer are primarily determined by the 7th edition of the Cancer Staging Manual by the American Joint Committee on Cancer (AJCC 7th), as well as the staging guidelines provided by the International Federation of Gynecology and Obstetrics in 2018 (Bhatla and Denny 2018). However, increasing evidence suggests that those who share a similar stage of cervical cancer may exhibit varying prognoses, particularly individuals with large tumor size or those diagnosed with endocervical adenocarcinoma (Farley et al. 2003; Xie et al. 2018). Nomograms are assessment tools that quantify risks and benefits, allowing clinicians to predict the occurrence and progression of disease based on meaningful clinical indicators. Nomograms have found extensive application in anticipating the prognosis of individuals with diverse cancer types, such as gastric cancer, lung cancer, and breast cancer (Pan et al. 2019; Yang et al. 2022; Yu and Zhang 2019). Herein, we established and verified the accuracy of nomogram models for anticipating the overall survival (OS) rate at 1, 3, and 5 years and the cancer-specific survival (CSS) rate for CA patients. These models can provide clinicians with more accurate and personalized prognosis estimates, helping them create reliable clinical treatment plans for CA patients.

## Methods

### Data source and patients

This study encompassed individuals who received a diagnosis of cervical adenocarcinoma during the period between 2004 and 2015. The data were procured from the SEER database of the National Cancer Institute employing SEER*Stat software (version 8.4.0.1; http://seer.cancer.gov/). The researchers acquired official authorization and explicit consent from the SEER program to access and utilize the data, ensuring that patient privacy was protected throughout the entire process.

**Fig. 1:**
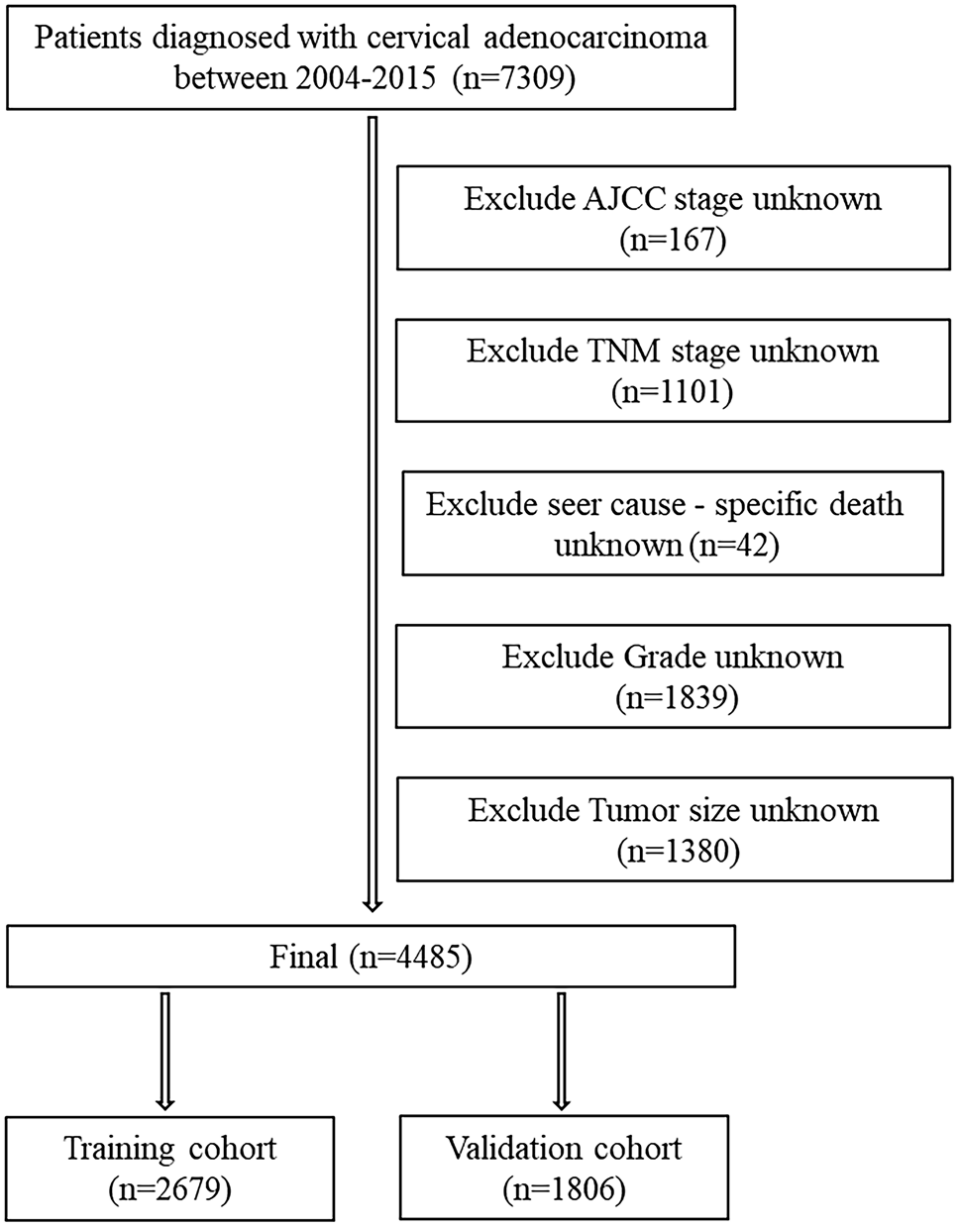
Flow chart for screening patients with cervical adenocarcinoma. *AJCC* American Joint Committee on Cancer; *TNM* tumor node metastasis stages

The following criteria were used for inclusion: (1) site recode ICDO-3/WHO2008: Cervix Uteri, (2) histologic type ICD-O-3 were: 8089/3, 8140/3, 8144/3, 8200/3, 8210/3, 8244/3, 8255/3, 8260/3, 8261/3, 8262/3, 8263/3, 8310/3, 8313/3, 8323/3, 8384/3, 8441/3, 8460/3, 8480/3, 8481/3, 8482/3, 8574/3, (3) year of diagnosis: 2004–2015, (4) known CS tumor size (2004–2015), (5) known reason for death and the duration of survival. The following criteria were used for exclusion: (1) unknown survival time; (2) unknown AJCC stage; (3) unknown TNM stage; (4) unknown seer cause-specific death; (5) unknown grade; (6) unidentified tumor dimensions. The screening flowchart for the subjects is depicted in Fig. 1. Overall, 4485 patients were assigned to a training cohort (*n* = 2679) and testing cohort (*n* = 1806) in a 3:2 ratio.

### Study variables

The SEER database provided clinical variables for this study, including age at diagnosis, race, histologic type, TNM stage, AJCC stage, chemotherapy, radiotherapy, histologic grade, tumor size (in cm), classification of cause-specific mortality, recoding of vital status, and the duration of survival. Age was subjectively categorized as less than 30, 30–50, 50–70, or 70 or older. Tumor variables included radiotherapy (yes or no/unknown), chemotherapy (yes or no/unknown), and tumor size (in cm) (< 2 cm, 2–4 cm, or 4 cm or greater). Tumor grades I-IV were further classified as well differentiated, moderately differentiated, poorly differentiated, or undifferentiated.

The primary outcome measure of this study was overall survival (OS), while the secondary outcome measure was cancer-specific survival (CSS). OS time refers to the length of time a patient survives from diagnosis until death from any cause. If a patient was lost to follow-up, their last recorded time was typically used as their time of death. CSS time, on the other hand, represents the length of time a patient survives with cancer from diagnosis until cancer-related death, excluding other factors that may have contributed to their death.

### Statistical analysis

Categorical variables were presented as frequencies (*n*) and percentages (%). Univariate and multivariate Cox analyses were performed to identify independent prognostic determinants and develop nomograms for assessing overall survival (OS) and cancer-specific survival (CSS) in the training cohort, respectively. Variables displaying *P*-values less than 0.05 in both the univariate and multivariate analyses were deemed statistically significant. The Cox analysis was performed using SPSS software (version 25.0, Chicago, IL, USA).

The prognostic nomograms were developed through LASSO regression, utilizing the findings of the multivariate Cox analysis in the training cohort. To assess the prognostic accuracy of the model, the concordance index (C-index) and calibration curve were employed. Furthermore, the predictive performance of the nomograms for 1, 3, and 5-year OS was assessed employing the receiver operating characteristic (ROC) curve. The R version 4.2.1 software in R Studio was used to construct the nomograms, C-index, calibration curves, and ROC curves.

## Results

### Patient baseline characteristics

In this study, a total of 4485 patients with cervical adenocarcinoma from 2004–2015 were identified in the SEER database. Subsequently, the entire dataset was randomly classified into a training cohort (*n* = 2679) and a testing cohort (*n* = 1806) in a 3:2 ratio. For all patients, the count of patients categorized by age < 30, 30–50, 50–70, and ≥ 70 years old was 216 (4.82%), 2520 (56.19%), 1387 (30.93%), and 362 (8.07%), respectively. For the race group, 3708 (82.68%) patients were white, 255 (5.69%) patients were black, and 522 (11.64%) patients were others. The total count of patients in AJCC stage I, II, III, and IV were 3047 (67.94%) 442 (9.86%), 645 (14.38%), and 351 (7.83%), respectively. The total count of patients with chemotherapy was 1617 (36.05%) and 2868 (63.95%). The total count of patients with radiotherapy was 1953 (43.55%) and 2532 (56.45%). The total count of patients in tumor grade I, II, III, and IV were 1425 (31.77%), 1851 (41.27%), 1056 (23.55%), and 153 (3.41%), respectively. Total count of patients with tumor size < 2 cm, 2–4 cm, and ≥ 4 cm were 1790 (39.91%), 1284 (28.63%), and 1411 (31.46%). For all the variables examined in both the training and testing cohorts, the chi-square test yielded results with *P*-values greater than 0.05. Table 1 displays the baseline features of the participants enrolled.

**Table 1:** The demographics and clinical characteristics of patients in training cohort and testing cohort

Characteristic	Total (*N* = 4485)	Training (*N* = 2679)	Testing (*N* = 1806)	*P* value
Age, years old, no. (%)				0.194
< 30	216 (4.82%)	133 (4.96%)	83 (4.60%)	
30–49	2520 (56.19%)	1527 (57.00%)	993 (55.00%)	
50–69	1387 (30.93%)	820 (30.6%)	567 (31.4%)	
≥70	362 (8.07%)	199 (7.43%)	163 (9.03%)	
Race, no. (%)				0.751
White	3708 (82.68%)	2221 (82.9%)	1487 (82.3%)	
Black	255 (5.69%)	154 (5.75%)	101 (5.59%)	
Others	522 (11.64%)	304 (11.3%)	218 (12.1%)	
Marriage				0.677
Single	1013 (22.59%)	608 (22.7%)	405 (22.4%)	
Unmarried or domestic partner	2491 (55.54%)	1497 (55.9%)	994 (55.0%)	
Married	981 (21.87%)	574 (21.4%)	407 (22.5%)	
T stage, no. (%)				0.522
T1	3425 (76.37%)	2063 (77.0%)	1362 (75.4%)	
T2	704 (15.70%)	404 (15.1%)	300 (16.6%)	
T3	289 (6.44%)	174 (6.49%)	115 (6.37%)	
T4	67 (1.49%)	38 (1.42%)	29 (1.61%)	
N stage, no. (%)				0.647
N0	3755 (83.72%)	2249 (83.9%)	1506 (83.4%)	
N1	730 (16.28%)	430 (16.1%)	300 (16.6%)	
M stage, no. (%)				0.444
M0	4167 (92.91%)	2496 (93.2%)	1671 (92.5%)	
M1	318 (7.09%)	183 (6.83%)	135 (7.48%)	
AJCC stage, no. (%)				0.533
I	3047 (67.94%)	1841 (68.7%)	1206 (66.8%)	
II	442 (9.86%)	259 (9.67%)	183 (10.1%)	
III	645 (14.38%)	379 (14.1%)	266 (14.7%)	
IV	351 (7.83%)	200 (7.47%)	151 (8.36%)	
SEER stage, no. (%)				0.384
Localized	2926 (65.24%)	1766 (65.9%)	1160 (64.2%)	
Regional	1202 (26.80%)	710 (26.5%)	492 (27.2%)	
Distant	357 (7.96%)	203 (7.58%)	154 (8.53%)	
Grade, no. (%)				0.213
I	1425 (31.77%)	861 (32.1%)	564 (31.2%)	
II	1851 (41.27%)	1105 (41.2%)	746 (41.3%)	
III	1056 (23.55%)	634 (23.7%)	422 (23.4%)	
IV	153 (3.41%)	79 (2.95%)	74 (4.10%)	
Tumor size, cm, no. (%)				0.896
< 2	1790 (39.91%)	1073 (40.1%)	717 (39.7%)	
2–4	1284 (28.63%)	760 (28.4%)	524 (29.0%)	
≥ 4	1411 (31.46%)	846 (31.6%)	565 (31.3%)	
Initial chemotherapy, no. (%)				0.931
Yes	2868 (63.95%)	1715 (64.0%)	1153 (63.8%)	
No/unknown	1617 (36.05%)	964 (36.0%)	653 (36.2%)	
Initial radiotherapy, no. (%)				0.709
Yes	2532 (56.45%)	1519 (56.7%)	1013 (56.1%)	
No/unknown	1953 (43.55%)	1160 (43.3%)	793 (43.9%)	

### Exploring independent prognosis-predictive factors of OS within the training set

In the univariate analysis, all variables except for “unmarried or domestic partner” in the marriage group were found to be significant regarding overall survival (OS), as shown in Table 2. In the multivariate analysis for OS, ten variables—age, race, AJCC TNM stage, SEER stage, chemotherapy, radiotherapy, grade, and tumor size—were found to be independent prognostic factors. LASSO regression was utilized to create a predictive model using 10 variables from the multivariate analysis of the training set. Ultimately, seven variables—age, AJCC TNM stage, SEER stage, grade, and tumor size—were considered statistically significant factors and used to establish a nomogram that predicts 1-, 3-, and 5-year OS.

**Table 2:** Association between variables and overall survival

Characteristic	Univariate HR	Multivariate HR
Age, years old, no. (%)		
< 30	Reference	Reference
30–49	1.03 (0.65–1.63)	1.13 (0.71–1.80)
50–69	2.73 (1.73–4.29)	1.78 (1.12–2.85)
≥70	7.35 (4.60–11.76)	4.50 (2.74–7.39)
Race, no. (%)		
White	Reference	Reference
Black	2.60 (2.04–3.30)	1.83 (1.43 to2.34)
Others	1.42 (1.14–1.77)	1.26 (1.00–1.57)
Marriage		
Single	Reference	Reference
Unmarried or domestic partner	0.84 (0.69 to 1.02)	0.90 (0.74 to 1.11)
Married	1.71 (1.39 to 2.11)	1.07 (0.86 to 1.35)
T stage, no. (%)		
T1	Reference	Reference
T2	4.33 (3.62–5.17)	1.49 (1.11–2.02)
T3	12.00 (9.78–14.71)	3.11 (2.33–4.14)
T4	21.29 (14.79–30.64)	4.41 (2.61–7.47)
N stage, no. (%)		
N0	Reference	Reference
N1	4.14 (3.54–4.84)	1.39 (1.07–1.81)
M stage, no. (%)		
M0	Reference	Reference
M1	8.15 (6.75–9.83)	3.26 (1.46–7.27)
AJCC stage, no. (%)		
I	Reference	Reference
II	4.52 (3.60–5.67)	0.81 (0.47–1.40)
III	5.99 (4.94–7.27)	0.86 (0.51–1.46)
IV	16.24 (13.18–20.01)	0.55 (0.23–1.33)
SEER stage, no. (%)		
Localized	Reference	Reference
Regional	5.39 (4.48–6.38)	1.92 (1.25–2.97)
Distant	16.50 (13.33–20.42)	2.64 (1.27–5.49)
Grade, no. (%)		
I	Reference	Reference
II	1.83 (1.46–2.28)	1.30 (1.04–1.63)
III	4.22 (3.39–5.24)	1.66 (1.32–2.09)
IV	4.77 (3.30–6.88)	1.59 (1.08–2.33)
Tumor size, cm, no. (%)		
< 2	Reference	Reference
2–4	2.40 (1.88–3.07)	1.44 (1.11–1.88)
≥4	7.08 (5.71–8.79)	2.48 (1.90–3.23)
Initial chemotherapy, no. (%)		
Yes	3.62 (3.10–4.23)	0.68 (0.55–0.85)
No/unknown	Reference	Reference
Initial radiotherapy, no. (%)		
Yes	3.70 (3.14–4.36)	1.39 (1.12–1.72)
No/unknown	Reference	Reference

### Construction of prognostic nomograms

**Fig. 2:**
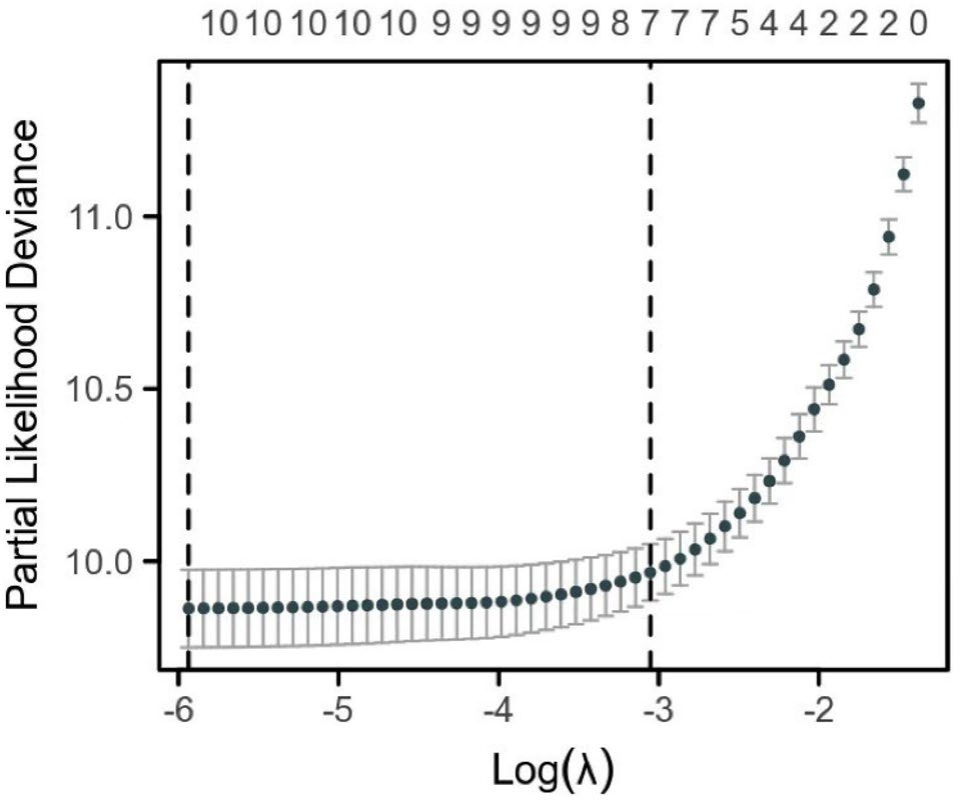
Selection of significant parameters in clinicopathologic variables in the training set and definition of linear predictor. Seven time cross-validation for tuning parameter selection in the LASSO model. The LASSO was used for regression of high dimensional predictors. The method uses an L1 penalty to shrink some regression coefficients to exactly zero. The binomial deviance curve was plotted versus log (λ), where λ is the tuning parameter. *LASSO* least absolute shrinkage and selection operator

**Fig. 3:**
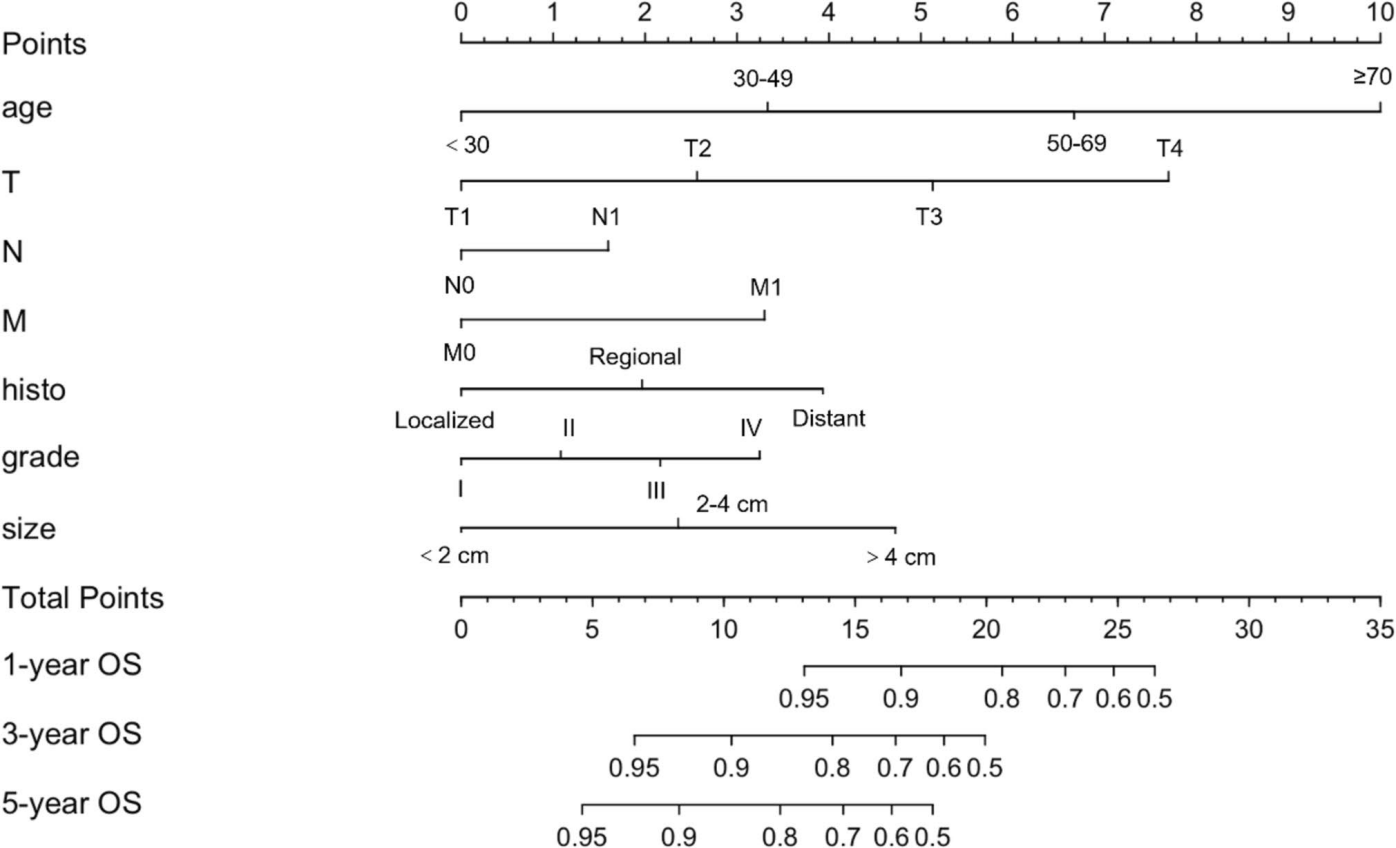
Nomograms for predicting 1-, 3- and 5-year OS in training cohort. grade: I, well differentiated; II, moderately differentiated; III, poorly differentiated, IV, undifferentiated or anaplastic; *OS* overall survival

Nomograms were developed to predict 1, 3, and 5-year survival for OS in the training set based on statistically significant prognostic factors identified through LASSO regression analysis (Figs. 2 and 3). The nomogram computes a predicted score for each variable by its corresponding point and then sums up all the scores of the variables to obtain the total score. To assess the anticipated probability of 1, 3, and 5-year OS for individual patients, a straight line is drawn from the “total point” to the respective “survival axes”. Age and T stage were found to be more important predictive factors than other variables in the OS nomogram.

### Validation and calibration of the nomogram for OS and CSS

**Fig. 4:**
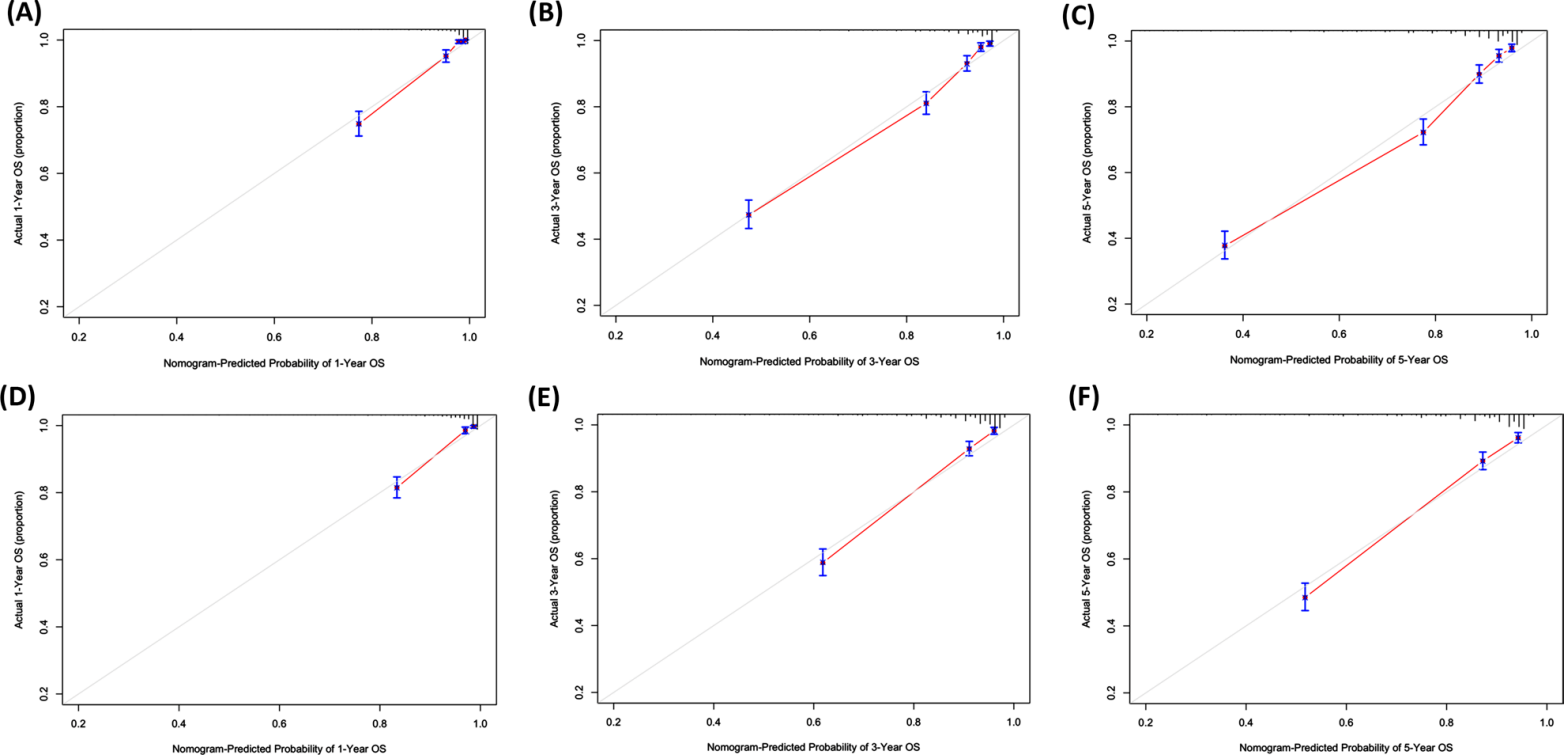
Calibration plots of 1-, 3-, and 5-year OS prediction in training cohort (**A**, **B**, **C**) and testing cohort (**D**, **E**, **F**)

The nomogram was validated using the C-index. Figure 4 demonstrates that the nomogram predicted OS with a C-index of 0.832 (95% CI, 0.817–0.847) in the training cohort and a C-index of 0.823 (95% CI, 0.805–0.841) in the testing cohort. These findings imply that the nomogram exhibits potential as a promising tool for anticipating OS in patients. Calibration plots were constructed to compare the performance of the nomogram with an ideal curve, and the outcomes demonstrated strong concordance between the predicted values from the nomogram and the actually observed values in both the training and testing cohorts.

**Fig. 5:**
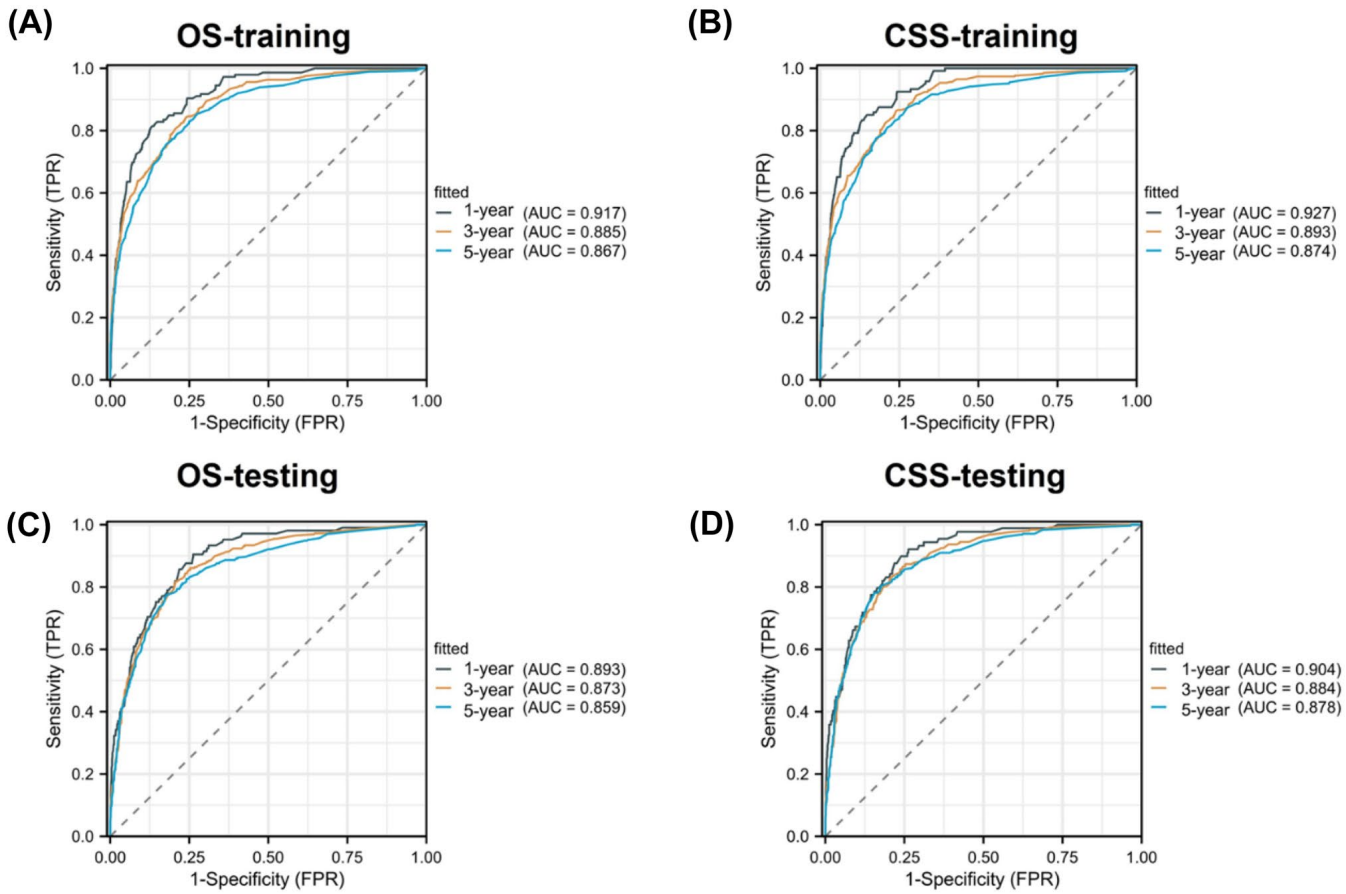
ROC curves verified the predictive value of nomogram. ROC of 1-, 3- and 5-year OS in the training cohort (**A**) and testing cohort (**C**). ROC of 1-, 3- and 5-year CSS in the training cohort (**B**) and testing cohort (**D**)

Furthermore, we evaluated the performance of the model in predicting overall survival (OS) in the testing cohort and assessed its predictive value for cancer-specific survival (CSS). In the training cohort, the receiver operating characteristic (ROC) curve's area under the curve (AUC) values for 1, 3, and 5-year overall survival (OS) were recorded to be 0.917, 0.885, and 0.867, respectively. Similarly, in the testing cohort, the corresponding AUC values were 0.893, 0.873, and 0.859. Additionally, the AUC values for CSS at 1, 3, and 5 years were 0.927, 0.893, and 0.874 in the training set, and 0.904, 0.884, and 0.878 in the testing set, respectively (Fig. 5).

### Clinical applicability of the nomogram

**Fig. 6:**
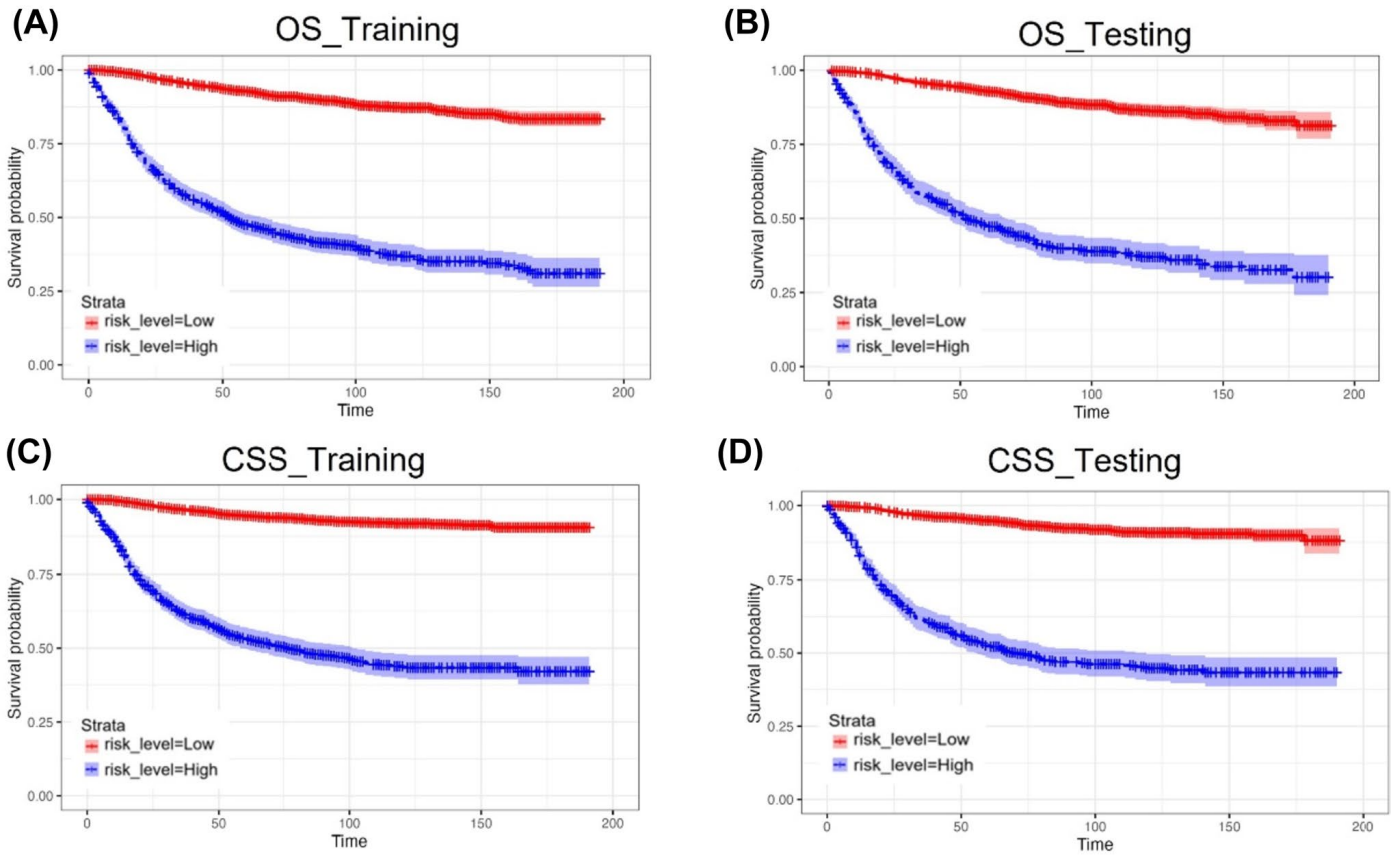
Kaplan–Meier curves of OS and CSS in the training cohort (**A**, **C**) and testing cohort (**B**, **D**)

**Fig. 7:**
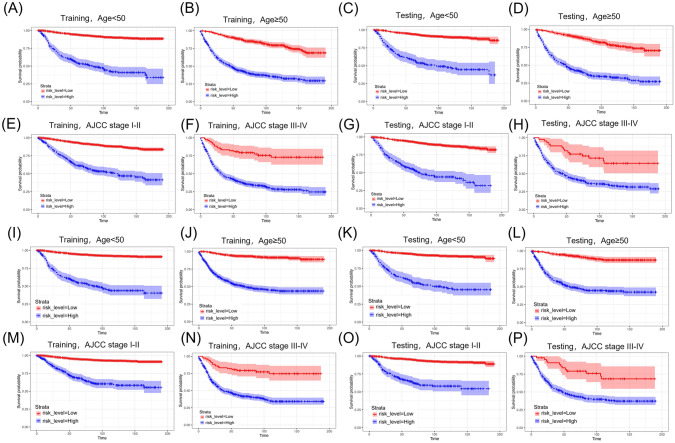
Kaplan–Meier curves of OS and CSS based on age in the training cohort (**A**, **B**, **I**, **J**) and testing cohort (**C**, **D**, **K**, **L**). Kaplan–Meier curves of OS and CSS based on AJCC stage in the training cohort (**E**, **F**, **M**, **N**) and testing cohort (**G**, **H**, **O**, **P**)

To assess the practicality and effectiveness of the prognosis-predictive nomogram, patients were classified into two groups: high-risk and low-risk, as per their respective prognostic scores. The optimal cutoff value was determined by selecting the maximum Youden index at 5 years using the ROC curve. Kaplan–Meier survival analysis for OS and CSS was performed for both training and testing cohorts, and a significant difference in prognosis was detected between the two groups. The results indicated that patients with high-risk prognostic scores had significantly reduced OS and CSS in both the training and testing cohorts (*P* < 0.05) (Fig. 6). Furthermore, age and AJCC stage were selected as important clinical prognostic factors and were categorized into two groups, namely high-risk and low-risk, according to their respective prognostic scores for further validation. The survival curves of different age and AJCC stage groups showed similar trends (*P* < 0.05) (Fig. 7). The median survival of OS and CSS between high-risk and low-risk groups was shown in Table S1 and Table S2.

## Discussion

Cervical cancer is a prevalent form of malignant tumor in women, ranking fourth in the incidence of female malignant tumors (Small et al. 2017; Johnson et al. 2019). On a global scale, the annual incidence of cervical cancer surpasses 500,000 cases among women, with a corresponding mortality toll of over 300,000 individuals succumbing to this disease (Cohen et al. 2019). It continues to be one of the primary burdens of cancer worldwide. While cervical cancer screening and HPV vaccination have reduced the incidence in many countries, the incidence of cervical adenocarcinoma has been steadily increasing (Bray et al. 2005; de Martel et al. 2017). Moreover, patients with adenocarcinoma tend to be younger than those with squamous cell carcinoma, and there is mounting evidence that they are more likely to still be in the reproductive stage (Loureiro and Oliva 2014). The rise in adenocarcinoma incidence is not entirely clear, but it may relate to the internal growth pattern of endocervical glands, which hinders accurate measurement of invasion depth and diagnosis (Rivera-Colon et al. 2020). The FIGO staging system remains an essential standard for determining cervical cancer treatment. Patients with the same clinical stage may exhibit different behaviors and survival outcomes due to different histologic subtypes. In 2013, Silva et al. proposed a new model for risk stratification of cervical adenocarcinoma based on interstitial invasion of the tumor (Silva classification) (Roma et al. 2015). This model is repeatable and better predicts the risk of lymph node metastasis compared with FIGO clinical staging criteria. However, Silva’s classification is still in the early stages and requires further clinicopathological studies to verify (Roma et al. 2015, 2016; Rutgers et al. 2016). Therefore, developing an individual prediction model for cervical adenocarcinoma is necessary to accurately predict prognosis and optimize treatment planning. In this study, we identified seven independent risk factors and provided a convenient nomogram prediction model to evaluate individual probabilities for the overall survival of cervical adenocarcinoma. We further explored this nomograph for cancer-specific survival in cervical adenocarcinoma.

According to the Cox analysis, the age of diagnosis and T stage are independent predictive variables for OS and they were also the top two contributing factors for OS in the nomograms. Our study reveals that as the grade of tumor T stage increases, the risk of poor prognosis also increases accordingly. In the nomograms, age contributes the most to the eventual risk score for OS (Fig. 2). Currently, the impact of age on the prognosis of cervical cancer individuals is a topic of debate. Some researchers support the conclusion that the risk of poor survival outcomes increases with age (Chen et al. 1999; Quinn et al. 2019; Sharma et al. 2012; Wright et al. 2005; Yagi et al. 2019). Older patients are less likely to receive aggressive treatment or may refuse it altogether. Conversely, other research indicates that younger patients experience less favorable prognoses and decreased survival rates, particularly in the advanced stages (Lau et al. 2009). One possible explanation is that young women have a significantly higher incidence of cervical adenocarcinoma (Lee et al. 2011; Loureiro and Oliva 2014; Sasieni and Adams 2001), which is harder to detect and has a high rate of missed diagnosis by cytology screening (Kiser and Butler 2020). The pathogenic factors behind this include infection by human papillomavirus (HPV) types 16, 18, and 45, oral contraceptive use, hormone replacement therapy, obesity, and others (Dahlström et al. 2010). Therefore, analyzing the prognosis of CA requires considering the factor of age. Radiotherapy and chemotherapy have also played an important role in the treatment of cervical cancer, thanks to advances in medical technology and treatment plans.

Table 2 shows that the use of radiotherapy and chemotherapy independently served as prognostic factors for OS. Interestingly, radiotherapy was found to result in a poor prognosis for OS. It could be inferred that the long-term survival of CA patients may be negatively affected by the side effects of radiotherapy, which can often be severe and occur several years after treatment (Bryant et al. 2017). Recent evidence suggests that neoadjuvant chemoradiotherapy followed by radical surgery is more effective for improving OS and PFS than concomitant chemotherapy and radiotherapy (CCRT) for locally advanced cervical adenocarcinoma (Tian et al. 2021). In a study conducted by Kazuhiro Suzuki et al., adjuvant CCRT following radical hysterectomy did not significantly improve survival in FIGO stage IIIC1 cervical adenocarcinoma patients (Suzuki et al. 2021). Another retrospective study found that while adjuvant radiotherapy decreased the recurrence rate from 44% to 9% in patients with adenocarcinomas and adenosquamous carcinomas, it did not provide any significant survival benefits (Galic et al. 2012). Although the concept of combining chemotherapy and radiotherapy is not new, the optimal chemotherapy regimen and sequence to deliver radiotherapy and chemotherapy remain unclear, especially for cervical adenocarcinoma (Benard et al. 2017). Therefore, new treatment approaches should be considered for this cancer type.

Nomograms were used to predict overall survival (OS) and other variables such as TNM stage, SEER stage, tumor grade, and tumor size were classified as independent risk factors impacting the prognosis of CA patients. In the United States, it has been observed that black women exhibit reduced survival rates than white women (Benard et al. 2017). TNM stage stands as the most prevalent tumor staging system globally and is determined by laboratory and postoperative pathological examinations. It is an independent prognostic factor for cervical HPV-related adenocarcinoma patients. However, the TNM stage has its limitations as it does not provide personalized prognosis prediction for a patient. Tumor grade has also been reported as an independent prognostic factor in cervical patients (Chung et al. 1981). Although there is a limited number of nomograms explicitly developed to anticipate OS and CSS among CA patients, this study evaluated many influencing factors, and accurate predictions can be made based on all significant factors included in the nomogram. The establishment of these nomograms will be useful in designing individualized treatments for CA patients.

There are several constraints to this research. Firstly, it was a retrospective study based on the SEER database, which makes it susceptible to selection biases that may affect the analysis results. As a result, more large-scale prospective studies are required to verify and explore potential prognostic factors. Secondly, the SEER database lacks information regarding specific treatments, such as endocrine therapy and chemotherapy programs, cycles, and doses. Due to these data limitations, family history and gene mutation status were unknown, in addition to HER-2 status, which could not be included in this study. Thirdly, there is a lack of external data to further validate the results of the competing risk model analysis and the predictive power of the nomogram. Therefore, we plan to utilize additional repositories in the United States and various countries to enhance the model during subsequent analyses.

## Conclusion

In this study, we established independent prognostic factors for cancer patients using the SEER database. We also constructed nomograms to predict 1, 3, and 5-year overall survival rates. The model demonstrated good predictive performance and may aid clinicians in evaluating survival prognosis and creating personalized treatment plans for cancer patients. However, because of the study’s retrospective nature, there is some degree of selection bias. Future research should focus on prospective studies to further investigate these findings.

## Supplementary Information

Below is the link to the electronic supplementary material.

Supplementary file1 (DOCX 22 KB)

## Data Availability

Publicly available datasets were analyzed in this study. This data can be found here: https://seer.cancer.gov.

## References

[CR1] Benard VB, Watson M, Saraiya M, Harewood R, Townsend JS, Stroup AM, Allemani C (2017) Cervical cancer survival in the United States by race and stage (2001–2009): findings from the concord-2 study. Cancer 123(Suppl 24):5119–5137. 10.1002/cncr.30906 29205300 10.1002/cncr.30906 PMC6386458

[CR01] Bhatla N, Denny L (2018) FIGO cancer report 2018. Int J Gynecol Obstet 143(Suppl 2):2–3. 10.1002/ijgo.12608 10.1002/ijgo.12608 30306587

[CR2] Bray F, Carstensen B, Møller H, Zappa M, Žakelj MP, Lawrence G, Weiderpass E (2005) Incidence trends of adenocarcinoma of the cervix in 13 European countries. Cancer Epidemiol Biomarkers Prev 14(9):2191–2199. 10.1158/1055-9965.EPI-05-0231 16172231 10.1158/1055-9965.EPI-05-0231

[CR3] Bryant AK, Banegas MP, Martinez ME, Mell LK, Murphy JD (2017) Trends in radiation therapy among cancer survivors in the united states, 2000–2030. Cancer Epidemiol Biomarkers Prev 26(6):963–970. 10.1158/1055-9965.EPI-16-1023 28096199 10.1158/1055-9965.EPI-16-1023

[CR4] Chen R-J, Lin Y-H, Chen C-A, Huang S-C, Chow S-N, Hsieh C-Y (1999) Influence of histologic type and age on survival rates for invasive cervical carcinoma in Taiwan. Gynecol Oncol 73(2):184–190. 10.1006/gyno.1999.5364 10329032 10.1006/gyno.1999.5364

[CR5] Chung CK, Stryker JA, Ward SP, Nahhas WA, Mortel R (1981) Histologic grade and prognosis of carcinoma of the cervix. Obstet Gynecol 57(5):636–642 7219913

[CR6] Cohen PA, Jhingran A, Oaknin A, Denny L (2019) Cervical cancer. Lancet 393(10167):169–182. 10.1016/S0140-6736(18)32470-X 30638582 10.1016/S0140-6736(18)32470-X

[CR7] Dahlström LA, Ylitalo N, Sundström K, Palmgren J, Ploner A, Eloranta S, Sparén P (2010) Prospective study of human papillomavirus and risk of cervical adenocarcinoma. Int J Cancer 127(8):1923–1930. 10.1002/ijc.25408 20473898 10.1002/ijc.25408 PMC2930102

[CR8] de Martel C, Plummer M, Vignat J, Franceschi S (2017) Worldwide burden of cancer attributable to hpv by site, country and hpv type. Int J Cancer 141(4):664–670. 10.1002/ijc.30716 28369882 10.1002/ijc.30716 PMC5520228

[CR9] Farley JH, Hickey KW, Carlson JW, Rose GS, Kost ER, Harrison TA (2003) Adenosquamous histology predicts a poor outcome for patients with advanced-stage, but not early-stage, cervical carcinoma. Cancer 97(9):2196–2202. 10.1002/cncr.11371 12712471 10.1002/cncr.11371

[CR10] Galic V, Herzog TJ, Lewin SN, Neugut AI, Burke WM, Lu YS, Wright JD (2012) Prognostic significance of adenocarcinoma histology in women with cervical cancer. Gynecol Oncol 125(2):287–291. 10.1016/j.ygyno.2012.01.012 22266551 10.1016/j.ygyno.2012.01.012

[CR11] Irie T, Kigawa J, Minagawa Y, Itamochi H, Sato S, Akeshima R, Terakawa N (2000) Prognosis and clinicopathological characteristics of ib-iib adenocarcinoma of the uterine cervix in patients who have had radical hysterectomy. Eur J Surg Oncol 26(5):464–467. 10.1053/ejso.1999.0923 11016467 10.1053/ejso.1999.0923

[CR12] Johnson CA, James D, Marzan A, Armaos M (2019) Cervical cancer: an overview of pathophysiology and management. Semin Oncol Nurs 35(2):166–174. 10.1016/j.soncn.2019.02.003 30878194 10.1016/j.soncn.2019.02.003

[CR13] Kiser LH, Butler J (2020) Improving equitable access to cervical cancer screening and management. Am J Nurs 120(11):58–67. 10.1097/01.Naj.0000721944.67166.17 33105224 10.1097/01.NAJ.0000721944.67166.17

[CR14] Lau H-Y, Juang C-M, Chen Y-J, Twu N-F, Yen M-S, Chao K-C (2009) Aggressive characteristics of cervical cancer in young women in Taiwan. Int J Gynaecol Obstet 107(3):220–223. 10.1016/j.ijgo.2009.07.029 19716131 10.1016/j.ijgo.2009.07.029

[CR15] Lee Y-Y, Choi CH, Kim T-J, Lee J-W, Kim B-G, Lee J-H, Bae D-S (2011) A comparison of pure adenocarcinoma and squamous cell carcinoma of the cervix after radical hysterectomy in stage ib–iia. Gynecol Oncol 120(3):439–443. 10.1016/j.ygyno.2010.11.022 21145099 10.1016/j.ygyno.2010.11.022

[CR16] Loureiro J, Oliva E (2014) The spectrum of cervical glandular neoplasia and issues in differential diagnosis. Arch Pathol Lab Med 138(4):453–483. 10.5858/arpa.2012-0493-RA 24678677 10.5858/arpa.2012-0493-RA

[CR17] Pan X, Yang W, Chen Y, Tong L, Li C, Li H (2019) Nomogram for predicting the overall survival of patients with inflammatory breast cancer: a SEER-based study. Breast 47:56–61. 10.1016/j.breast.2019.05.015 31351202 10.1016/j.breast.2019.05.015

[CR18] Quinn BA, Deng X, Colton A, Bandyopadhyay D, Carter JS, Fields EC (2019) Increasing age predicts poor cervical cancer prognosis with subsequent effect on treatment and overall survival. Brachytherapy 18(1):29–37. 10.1016/j.brachy.2018.08.016 30361045 10.1016/j.brachy.2018.08.016 PMC6338515

[CR19] Rivera-Colon G, Chen H, Niu S, Lucas E, Holloway S, Carrick K, Zheng W (2020) Cervical adenocarcinoma: Histopathologic features from biopsies to predict tumor behavior. Am J Surg Pathol 44(2):247–254. 10.1097/PAS.0000000000001379 31567190 10.1097/PAS.0000000000001379

[CR20] Roma AA, Diaz De Vivar A, Park KJ, Alvarado-Cabrero I, Rasty G, Chanona-Vilchis JG, Silva EG (2015) Invasive endocervical adenocarcinoma: a new pattern-based classification system with important clinical significance. Am J Surg Pathol 39(5):667–672. 10.1097/pas.0000000000000402 25724003 10.1097/PAS.0000000000000402

[CR21] Roma AA, Mistretta T-A, De Vivar AD, Park KJ, Alvarado-Cabrero I, Rasty G, Silva EG (2016) New pattern-based personalized risk stratification system for endocervical adenocarcinoma with important clinical implications and surgical outcome. Gynecol Oncol 141(1):36–42. 10.1016/j.ygyno.2016.02.028 27016227 10.1016/j.ygyno.2016.02.028 PMC5068220

[CR22] Rutgers JKL, Roma AA, Park KJ, Zaino RJ, Johnson A, Alvarado I, Silva EG (2016) Pattern classification of endocervical adenocarcinoma: reproducibility and review of criteria. Mod Pathol 29(9):1083–1094. 10.1038/modpathol.2016.94 27255163 10.1038/modpathol.2016.94 PMC5506840

[CR23] Sasieni P, Adams J (2001) Changing rates of adenocarcinoma and adenosquamous carcinoma of the cervix in england. Lancet 357(9267):1490–1493. 10.1016/S0140-6736(00)04646- 11377601 10.1016/S0140-6736(00)04646-8

[CR24] Sharma C, Deutsch I, Horowitz DP, Hershman DL, Lewin SN, Lu Y-S, Wright JD (2012) Patterns of care and treatment outcomes for elderly women with cervical cancer. Cancer 118(14):3618–3626. 10.1002/cncr.26589 22038773 10.1002/cncr.26589

[CR25] Siegel RL, Miller KD, Jemal A (2019) Cancer statistics. CA Cancer J Clin 69(1):7–34. 10.3322/caac.21551 30620402 10.3322/caac.21551

[CR26] Small W Jr, Bacon MA, Bajaj A, Chuang LT, Fisher BJ, Harkenrider MM, Gaffney DK (2017) Cervical cancer: a global health crisis. Cancer 123(13):2404–2412. 10.1002/cncr.30667 28464289 10.1002/cncr.30667

[CR27] Suzuki K, Nagao S, Narita M, Nakazawa H, Shibutani T, Yamamoto K, Yamaguchi S (2021) Survival impact of adjuvant concurrent chemoradiotherapy after radical hysterectomy in figo stage iiic1 cervical adenocarcinoma. Int J Clin Oncol 26(7):1322–1329. 10.1007/s10147-021-01904-0 33825085 10.1007/s10147-021-01904-0

[CR28] Tian T, Gao X, Ju Y, Ding X, Ai Y (2021) Comparison of the survival outcome of neoadjuvant therapy followed by radical surgery with that of concomitant chemoradiotherapy in patients with stage ib2-iiib cervical adenocarcinoma. Arch Gynecol Obstet 303(3):793–801. 10.1007/s00404-020-05826-6 33009996 10.1007/s00404-020-05826-6

[CR29] Wright JD, Gibb RK, Geevarghese S, Powell MA, Herzog TJ, Mutch DG, Rader JS (2005) Cervical carcinoma in the elderly: an analysis of patterns of care and outcome. Cancer 103(1):85–91. 10.1002/cncr.20751 15540239 10.1002/cncr.20751

[CR30] Xie X, Song K, Cui B, Jiang J, Yang X, Kong B (2018) A comparison of the prognosis between adenocarcinoma and squamous cell carcinoma in stage ib–iia cervical cancer. Int J Clin Oncol 23(3):522–531. 10.1007/s10147-017-1225-8 29299705 10.1007/s10147-017-1225-8

[CR31] Yagi A, Ueda Y, Kakuda M, Tanaka Y, Ikeda S, Matsuzaki S, Kimura T (2019) Epidemiologic and clinical analysis of cervical cancer using data from the population-based Osaka cancer registry. Cancer Res 79(6):1252–1259. 10.1158/0008-5472.CAN-18-3109 30635276 10.1158/0008-5472.CAN-18-3109

[CR32] Yang Y, Shen C, Shao J, Wang Y, Wang G, Shen A (2022) Based on the development and verification of a risk stratification nomogram: predicting the risk of lung cancer-specific mortality in stage iiia-n2 unresectable large cell lung neuroendocrine cancer compared with lung squamous cell cancer and lung adenocarcinoma. Front Oncol. 10.3389/fonc.2022.825598 35847910 10.3389/fonc.2022.825598 PMC9282874

[CR33] Yu C, Zhang Y (2019) Development and validation of prognostic nomogram for young patients with gastric cancer. Ann Transl Med 7(22):641. 10.21037/atm.2019.10.77 31930042 10.21037/atm.2019.10.77 PMC6944578

