# Supplementary material for: Enhancing prognostic accuracy: a SEER-based analysis for overall and cancer-specific survival prediction in cervical adenocarcinoma patients
Source: J Cancer Res Clin Oncol. 2023 Sep 25;149(19):17027–37. doi: 10.1007/s00432-023-05399-2 (PMC10657287; doi:10.1007/s00432-023-05399-2)
Supplement: Supplementary file 1 — Supplementary file1 (DOCX 22 KB) [file 432_2023_5399_MOESM1_ESM.docx]

**Enhancing Prognostic Accuracy: A SEER-based Analysis for Overall and Cancer-Specific Survival Prediction in Cervical Adenocarcinoma Patients**

**Table S1. The median survival of OS and CSS between high risk and low risk groups in the training and testing cohort**

|  | | Overall survival | | Cancer-specific survival | |
| --- | --- | --- | --- | --- | --- |
|  |  | High risk | Low risk | High risk | Low risk |
| Training | Median survival (95%CI) | 53 (46-64) | NA | 77(58-101) | NA |
|  | HR (95%CI) | 8.03(7.87-8.19) | | 10.25(10.05-10.45) | |
|  | *P* | 2e-16 | | 2e-16 | |
| Testing | Median survival (95%CI) | 53(46-67) | NA | 71(56-119) | NA |
|  | HR (95%CI) | 7.87（7.67-8.06） | | 9.56（9.33-9.79） | |
|  | *P* | 2e-16 | | 2e-16 | |

**Table S2.** **The median survival of OS and CSS between high risk and low risk groups in the training and testing cohort based on age and AJCC stage.**

|  | | | Overall survival | | Cancer-specific survival | |
| --- | --- | --- | --- | --- | --- | --- |
|  |  |  | High risk | Low risk | High risk | Low risk |
| Training | Age<50 | Median survival (95%CI) | 83(58-112) | NA | 87(61-NA) | NA |
|  |  | HR (95%CI) | 8.90(8.66-9.15) | | 10.25(9.98-10.51) | |
|  |  | *P* | 2e-16 | | 2e-16 | |
|  | Age≥50 | Median survival (95%CI) | 46(37-54) | NA | 69(51-117) | NA |
|  |  | HR (95%CI) | 4.94(4.71-5.17) | | 9.25(8.91-9.60) | |
|  |  | *P* | 2e-16 | | 2e-16 | |
|  | Stage I-II | Median survival (95%CI) | 122 (82-NA) | NA | NA (164-NA) | NA |
|  |  | HR (95%CI) | 5.73(5.51-5.95) | | 7.09(6.83-7.36) | |
|  |  | *P* | 2e-16 | | 2e-16 | |
|  | Stage III-IV | Median survival (95%CI) | 32 (28-38) | NA | 38(31-53) | NA |
|  |  | HR (95%CI) | 4.24(3.78-4.70) | | 3.76(3.28-4.23) | |
|  |  | *P* | 1.01e-9 | | 5.52e-8 | |
| Testing | Age<50 | Median survival (95%CI) | 97(65-NA) | NA | NA | NA |
|  |  | HR (95%CI) | 7.74(7.44-8.04) | | 9.70(9.37-10.03) | |
|  |  | *P* | 2e-16 | | 2e-16 | |
|  | Age≥50 | Median survival (95%CI) | 46 (33-53) | NA | 59(46-115) | NA |
|  |  | HR (95%CI) | 5.40(5.12-5.68) | | 7.60(7.23-7.97) | |
|  |  | *P* | 2e-16 | | 2e-16 | |
|  | Stage I-II | Median survival (95%CI) | 75(59-141) | NA | NA (141-NA) | NA |
|  |  | HR (95%CI) | 7.40(7.14-7.66) | | 7.63(7.31-7.94) | |
|  |  | *P* | 2e-16 | | 2e-16 | |
|  | StageIII-IV | Median survival (95%CI) | 44 (32-55) | NA | 48(36-66) | NA |
|  |  | HR (95%CI) | 2.94(2.40-3.48) | | 3.06(2.47-3.64) | |
|  |  | *P* | 8.87e-5 | | 0.00017 | |
